# PARP Inhibitor PJ34 Suppresses Osteogenic Differentiation in Mouse Mesenchymal Stem Cells by Modulating BMP-2 Signaling Pathway

**DOI:** 10.3390/ijms161024820

**Published:** 2015-10-19

**Authors:** Yuta Kishi, Hisako Fujihara, Koji Kawaguchi, Hiroyuki Yamada, Ryoko Nakayama, Nanami Yamamoto, Yuko Fujihara, Yoshiki Hamada, Kazuhito Satomura, Mitsuko Masutani

**Affiliations:** 1 Department of Oral and Maxillofacial Surgery, School of Dental Medicine, Tsurumi University 2-1-3 Tsurumi, Tsurumi-ku, Yokohama, Kanagawa 230-8501, Japan; E-Mails: kishi-y@tsurumi-u.ac.jp (Y.K.); kawaguchi-k@tsurumi-u.ac.jp (K.K.); yamada-hi@tsurumi-u.ac.jp (H.Y.); yamamoto-nanami@tsurumi-u.ac.jp (N.Y.); hamada-y@tsurumi-u.ac.jp (Y.H.); 2 Department of Oral Hygiene, Tsurumi Junior College 2-1-3 Tsurumi, Tsurumi-ku, Yokohama, Kanagawa 230-8501, Japan; 3 Department of Pathology, School of Dental Medicine, Tsurumi University 2-1-3 Tsurumi, Tsurumi-ku, Yokohama, Kanagawa 230-8501, Japan; E-Mail: nakayama-r@tsurumi-u.ac.jp; 4 Department of Oral and Maxillofacial Surgery, Dentistry and Orthodontics, The University of Tokyo Hospital 7-3-1 Hongo, Bunkyo-ku, Tokyo 113-8655, Japan; E-Mail: fujiharay-ora@h.u-tokyo.ac.jp; 5 Department of Oral Medicine and Stomatology, School of Dental Medicine, Tsurumi University 2-1-3 Tsurumi, Tsurumi-ku, Yokohama, Kanagawa 230-8501, Japan; E-Mail: satomura-k@tsurumi-u.ac.jp; 6 Department of Frontier Life Science, Graduate School of Biochemical Science, Nagasaki University 1-7-1 Sakamoto, Nagasaki 852-8588, Japan; E-Mail: mmasutan@nagasaki-u.ac.jp; 7 Division of Chemotherapy and Clinical Cancer Research, National Cancer Center Research Institute 5-1-1 Tsukiji, Chuo-ku, Tokyo 104-0045, Japan; E-Mail: mmasutan@ncc.go.jp

**Keywords:** poly(ADP-ribosyl)ation, PARP inhibitor, mesenchymal stem cells, differentiation

## Abstract

Poly(ADP-ribosyl)ation is known to be involved in a variety of cellular processes, such as DNA repair, cell death, telomere regulation, genomic stability and cell differentiation by poly(ADP-ribose) polymerase (PARP). While PARP inhibitors are presently under clinical investigation for cancer therapy, little is known about their side effects. However, PARP involvement in mesenchymal stem cell (MSC) differentiation potentiates MSC-related side effects arising from PARP inhibition. In this study, effects of PARP inhibitors on MSCs were examined. MSCs demonstrated suppressed osteogenic differentiation after 1 µM PJ34 treatment without cytotoxicity, while differentiation of MSCs into chondrocytes or adipocytes was unaffected. PJ34 suppressed mRNA induction of osteogenic markers, such as *Runx2*, *Osterix*, *Bone Morphogenetic Protein-2*, *Osteocalcin*, *bone sialoprotein*, and *Osteopontin*, and protein levels of Bone Morphogenetic Protein-2, Osterix and Osteocalcin. PJ34 treatment also inhibited transcription factor regulators such as *Smad1*, *Smad4*, *Smad5* and *Smad8*. Extracellular mineralized matrix formation was also diminished. These results strongly suggest that PARP inhibitors are capable of suppressing osteogenic differentiation and poly(ADP-ribosyl)ation may play a physiological role in this process through regulation of BMP-2 signaling. Therefore, PARP inhibition may potentially attenuate osteogenic metabolism, implicating cautious use of PARP inhibitors for cancer treatments and monitoring of patient bone metabolism levels.

## 1. Introduction

Bone functions in a number of ways, including maintenance of organism structure, hematopoietic supply, mineral storage and so on. As the clinical importance of bone metabolism is high, protocols for osteogenic differentiation of mesenchymal stem cells (MSCs) are well established, with key markers for each differentiation step already identified [1,2,3,4]. During each step, required activation of specific transcription factors is controlled by factors such as bone morphogenetic protein (BMP), transforming growth factor-β (TGF-β), Wnt and hedgehog family proteins.

Post-transcriptional and post-translational modifications play an essential role in cellular processes and biological functions. In these processes, poly(ADP-ribosyl)ation is known to be involved in many cellular processes, such as DNA repair [5,6], cell death [7], telomere regulation [8], chromatin function and genomic stability [9]. Poly(ADP-ribosyl)ation is catalyzed by the poly(ADP-ribose) polymerase family (PARPs) using nicotinamide adenine dinucleotide (NAD) as a substrate to target proteins that lead to biological activities.

The most abundant PARP enzyme is PARP-1, whose deletion leads to increased sensitivity to anti-cancer drugs and ionizing radiation in mice [9,10]. PARP inhibitors also demonstrate sensitization to alkylating agents and ionizing radiation [11,12], and clinical trials for cancer therapy are now in progress [13]. Moreover, it was shown that *BRCA1/2*-mutated breast cancer had high sensitivity to PARP inhibitors in clinical trials [14]. The mechanism of action of PARP inhibitors is competitive blocking of NAD^+^ from binding to PARP-1 to synthesize polymer of ADP-ribose [15]. However, little is known about the side effects of PARP inhibitors except associated nausea, fatigue, and anemia. [16]. In recent years, the involvement of PARP family members in MSC differentiation has also been reported [17,18,19,20], including involvement in chondrogenic differentiation with PARP cleavage and activation of caspase-3 [20], as well as negative effects of PARP-2 on adipogenic differentiation [17]. Indirect regulation of osteogenic differentiation by PARP-1 through control of Tumor Necrosis Factor expression has also been demonstrated [18,19]. However, to our best knowledge, the function of PARP in BMP-2 signaling during osteogenic differentiation has not been clarified. Thus, we speculated that PARP activity might possibly be involved in regulation of MSC differentiation, suggesting possible side effects of PARP inhibitors on MSCs during and after cancer therapy.

In this study, we investigated the PARP inhibitors effects on proliferation and differentiation of two cell types. After determining PARP inhibitor concentrations demonstrating no cytotoxicity, the effects of PARP inhibitors on differentiation were analyzed. Our results would provide an understanding of biochemical osteogenic differentiation processes and theoretical basis for future clinical treatments using PARP inhibitors for cancer.

## 2. Results

### 2.1. Cytotoxicity Evaluation

**Figure 1:**
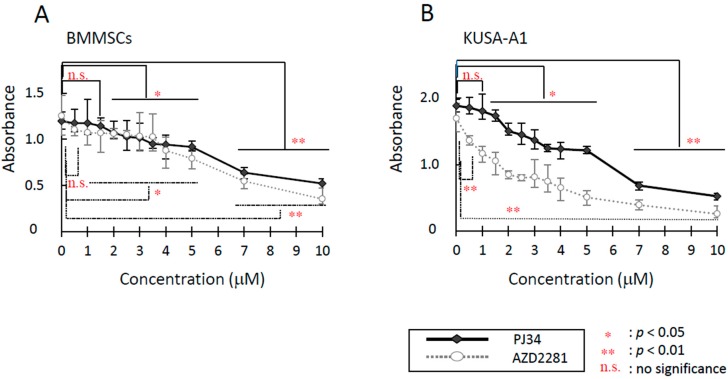
Cytotoxicity of PJ34 and AZD2281 on BMMSCs (**A**) and KUSA-A1 cells (**B**) were analyzed by survival assay. Cells were exposed to various concentrations of PARP inhibitors PJ34 and AZD2281 for 18 h, rinsed twice with PBS and allowed to grow for seven days. Values are expressed as mean ± SEM. * *p* < 0.05, ** *p* < 0.01, n.s. = no significance.

For the investigation of the cytotoxic effects of PARP inhibitors, PJ34 and AZD2281, on mouse bone marrow mesenchymal stem cells (BMMSCs) and mesenchymal progenitor cells (KUSA-A1 cells), two types of cytotoxic assays were performed. Inhibitors’ toxicity was concentration-dependent. In Microculture Tetrazolium Assay (MTT) assay, half maximal inhibitory concentration (IC_50_) for PJ34 on BMMSCs and KUSA-A1 cells after 24 h treatment was estimated at more than 10 µM. IC_50_ values for AZD2281 on BMMSCs and KUSA-A1 cells were both approximately 10 µM. However, cell viability was significantly reduced by PJ34 at 6 µM in BMMSCs and 4 µM in KUSA-A1. Viability was also significantly reduced by AZD2281 at 5 µM in BMMSCs and 3 µM in KUSA-A1 (Figure S1A,B). In survival assay, AZD2281 and a higher dose range of PJ34 were found to be cytotoxic for both cell types (Figure 1A,B). The cytotoxic effect of PJ34 was relatively mild and weaker than that of AZD2281, especially in KUSA-A1 cells. Concentrations of AZD2281 and PJ34 capable of suppressing cell survival by 50% were approximately 5.5 and 6.5 µM for BMMSCs, and 2 and 5 µM for KUSA-A1, respectively. From these results, 1–5 µM PJ34 was applied to assess PARP inhibitor effects with minimal cytotoxicity.

### 2.2. Effects of PJ34 on Cell Proliferation

**Figure 2:**
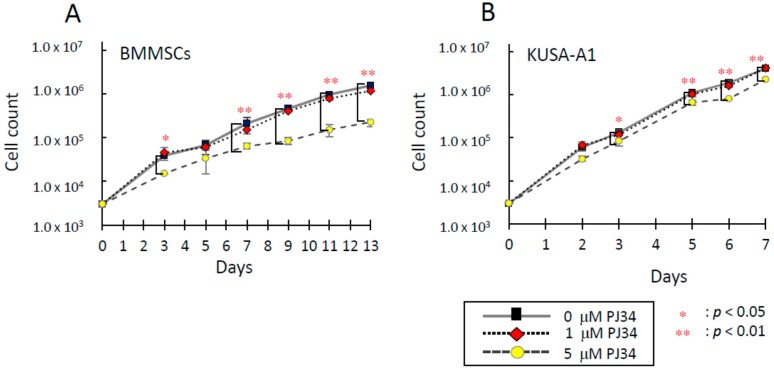
Effects of PJ34 on cell proliferation of BMMSCs (**A**) and KUSA-A1 (**B**) by proliferation assay. Values are expressed as mean ± SEM. * *p* < 0.05, ** *p* < 0.01.

Next, the effects of PJ34 on cell proliferation was analyzed. Dose-dependent suppressive effect of PJ34 on cell proliferation was exhibited (Figure 2A,B). Significant difference was not observed in the growth-rate of BMMSCs or KUSA-A1 cells cultured with 0 or 1 µM PJ34 during seven days, proving cells could maintain proliferation ability. However, cells cultured with 5 µM PJ34 showed significantly lower growth rates in both cell types.

### 2.3. Effects of PJ34 on Poly(ADP-ribosyl)ation

**Figure 3:**
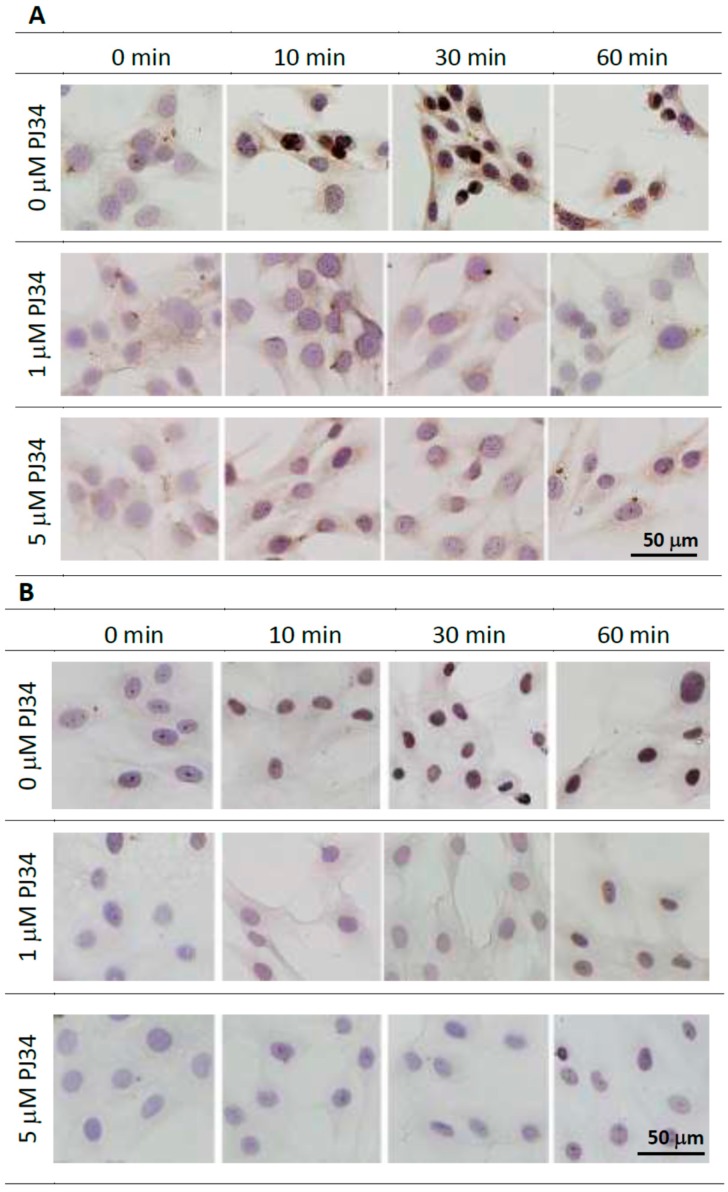
Inhibition of PARP activity in the presence of 1 and 5 µM PJ34 was confirmed by immunocytochemical analysis with anti-PAR antibody in BMMSCs (**A**) and KUSA-A1 cells (**B**), respectively. Scale bars = 50 µm.

To confirm that 1 and 5 µM PJ34 could effectively inhibit PARP activity, *i.e.*, poly(ADP-ribosyl)ation, we analyzed poly(ADP-ribose) (PAR) levels after treatment with hydrogen peroxide to stimulate DNA damage. Immunocytochemical staining of anti-PAR antibody indicated that PJ34 treatment significantly reduced PAR synthesis in response to the treatment of hydrogen peroxide (Figure 3A,B). PAR level was reduced in cells treated with either 1 or 5 µM PJ34 proving that the utilized dose of PJ34 could inhibit PARP activity in both cell types.

### 2.4. Effects of PJ34 on Osteogenic, Adipogenic and Chondrogenic Differentiation of MSCs

**Figure 4:**
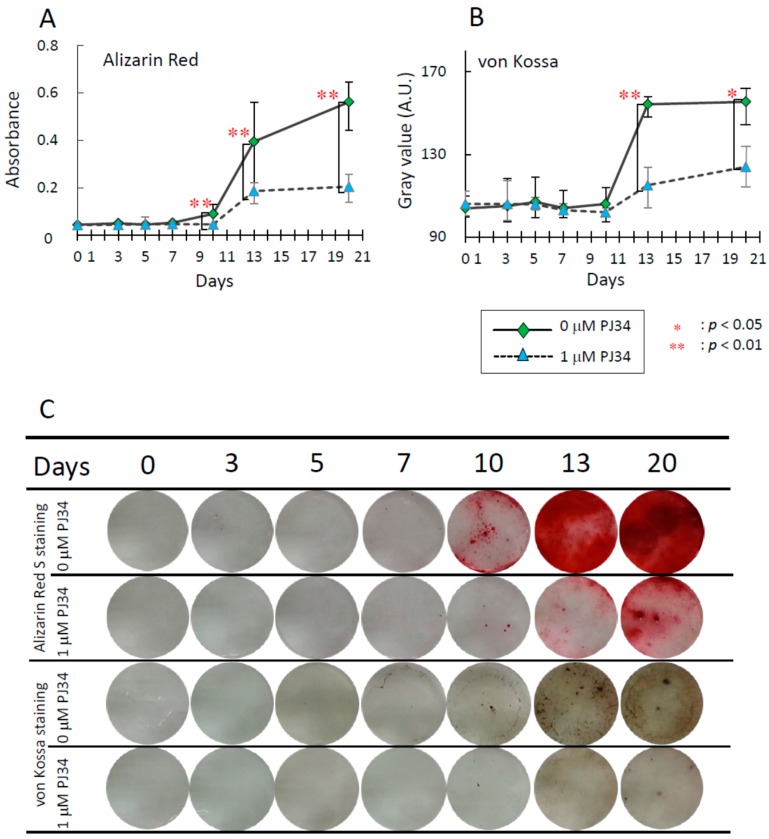
Effects of PJ34 on osteogenic differentiation of BMMSCs were analyzed by visualizing calcium deposition with Alizarin Red S staining (**A**) and deposition of calcium phosphate and calcium carbonate with von Kossa staining (**B**); (**C**) Representative photos of Alizarin Red S staining and von Kossa staining of BMMSCs during osteogenic differentiation. Values are expressed as mean ± SEM. A.U. = Arbitrary Unit. * *p* < 0.05, ** *p* < 0.01.

**Figure 5:**
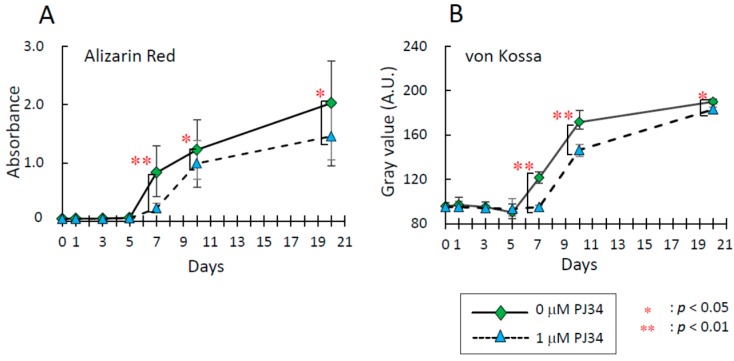

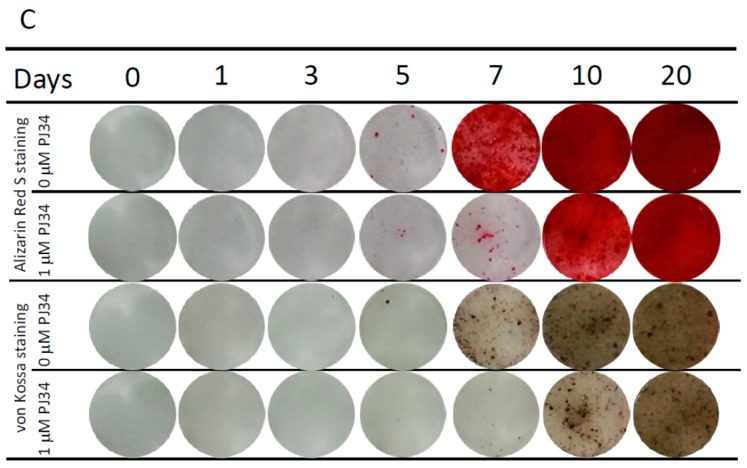
Effects of PJ34 on osteogenic differentiation of KUSA-A1 cells were also analyzed by Alizarin Red S staining (**A**) and von Kossa staining (**B**); (**C**) Representative photos of Alizarin Red S staining and von Kossa staining of KUSA-A1 cells during osteogenic differentiation. Values are expressed as mean ± SEM. A.U. = Arbitrary Unit. * *p* < 0.05, ** *p* < 0.01.

To investigate effects of the PARP inhibitor PJ34 on osteogenic differentiation of BMMSCs and KUSA-A1 cells, extracellular mineralized matrix formation was analyzed. Osteogenic differentiation status was determined by staining with Alizarin Red S for calcium deposition and von Kossa staining for deposition of calcium phosphate and calcium carbonate. The deposition level increased during osteogenic differentiation in time-dependent manner, however, these levels were significantly suppressed at each time point with 1 µM PJ34 treatment in BMMSCs (Figure 4A–C) and KUSA-A1 cells (Figure 5A–C).

**Figure 6:**
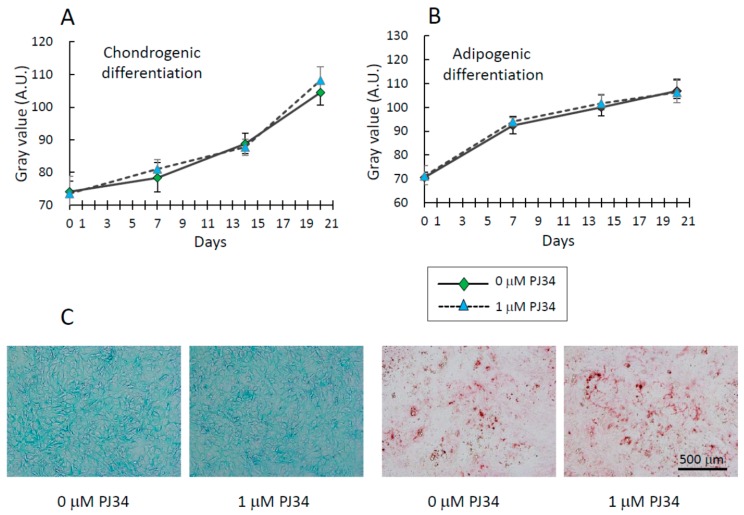
(**A**) Effects of PJ34 on chondrogenic differentiation of BMMSCs were analyzed by Alcian Blue staining.; (**B**) Effects of PJ34 on adipogenic differentiation of BMMSCs were analyzed by Oil Red O staining; (**C**) Representative photos of Alcian Blue staining (**Left**) and Oil Red O staining (**Right**) of BMMSCs 21 days differentiation into chondrogenic and adipogenic, respectively. Values are expressed as mean ± SEM. A.U. = Arbitrary Unit. Scale bar = 500 µm.

Chondrogenic and adipogenic differentiation of BMMSCs was analyzed using Alcian Blue staining and Oil Red O staining, respectively (Figure 6A–C). Only BMMSCs were analyzed since KUSA-A1 cells are mesenchymal progenitor cells, incapable of differentiating adipocyte or chondrocyte lineages [21]. During the examination period, almost the same level of staining was observed with or without 1 µM PJ34 in BMMSCs. Considering these differentiation patterns together, PJ34 appears to affect BMMSCs’ differentiation into osteoblasts, but not adipocytes or chondrocytes.

### 2.5. Effects of PJ34 on the Osteogenic Differentiation Markers

**Figure 7:**
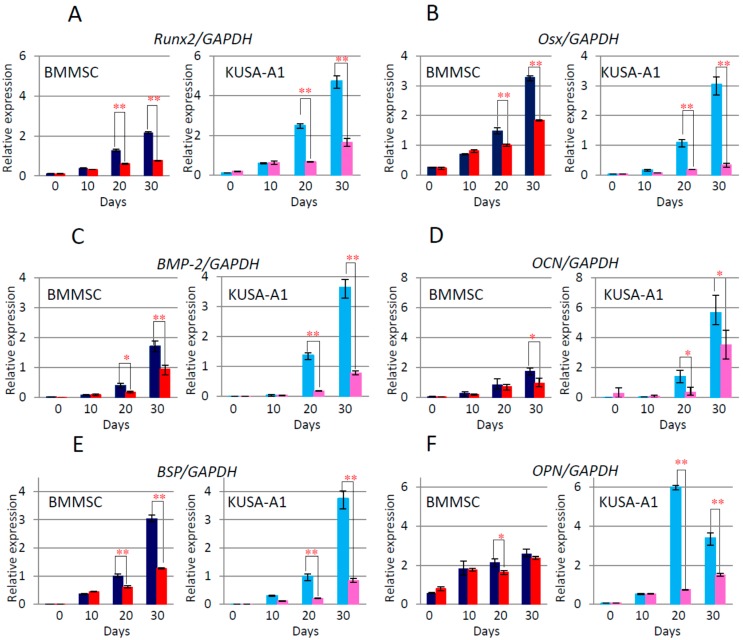

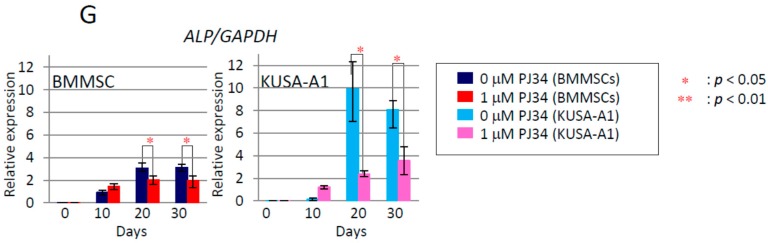
Effects of PJ34 on mRNA expression levels of osteogenic differentiation markers in BMMSCs and KUSA-A1 cells. Cells were treated with 0 and 1 µM PJ34 for 30 days, with medium changed every three days. The mRNA levels analyzed every 10 days were *Runx2* (**A**); *Osterix* (*Osx*) (**B**); *Bone Morphogenetic Protein-2* (*BMP-2*) (**C**); *Osteocalcin* (*OCN*) (**D**); *bone sialoprotein* (*BSP*) (**E**); *Osteopontin* (*OPN*) (**F**); and *alkaline phosphatase* (*ALP*) (**G**). Values are expressed as mean ± SEM. * *p* < 0.05, ** *p* < 0.01.

**Figure 8:**
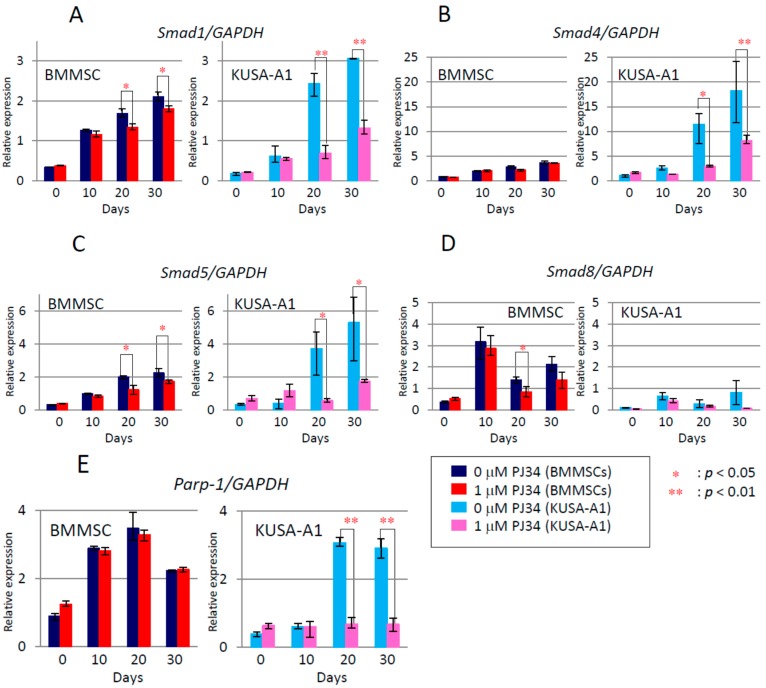
The effect of PJ34 on induction level of mRNA for transcription factors during osteogenic differentiation was also analyzed. Factors analyzed were *Smad1* (**A**); *Smad4* (**B**); *Smad5* (**C**); and *Smad 8* (**D**); Expression level of *Parp-1* was also analyzed (**E**). Values are expressed as mean ± SEM. * *p*< 0.05, ** *p* < 0.01.

By quantitative real-time PCR, we examined the effects of PJ34 on the expression of mRNA for osteogenic differentiation markers, *i.e.*, *Runx2*, *Osterix* (*Osx*), *Bone Morphogenetic Protein-2* (*BMP-2*), *Osteocalcin* (*OCN*), *bone sialoprotein* (*BSP*), and *Osteopontin* (*OPN*), and regulators of transcription factors, such as *Smad1*, *Smad4*, *Smad5* and *Smad8*. Additionally, *alkaline phosphatase* (*ALP*) and *Parp-1* expression were also examined. Time-dependent increase of the mRNA expression levels of these factors was observed during osteogenic differentiation, which was significantly attenuated following PJ34 treatment at days 20 and 30 in both cell types (Figure 7 and Figure 8). *Parp-1* expression was also decreased with 1 µM in KUSA-A1 cells, during the differentiation process. As a result, it was proved that the expressions of mRNA for these osteogenic marker genes and transcription factors were suppressed by PJ34.

### 2.6. Effects of PJ34 on Osteogenic Differentiation Marker Protein Levels

**Figure 9:**
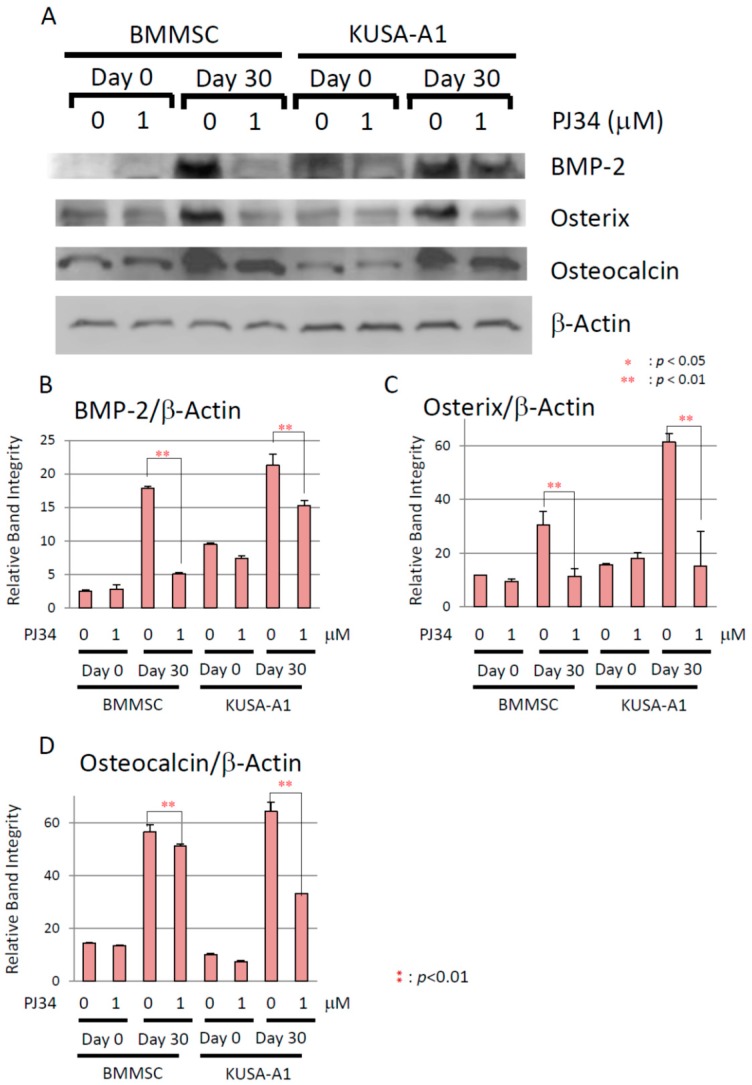
(**A**) The effect of PJ34 on protein levels for BMP-2 signaling pathway elements of BMMSCs and KUSA-A1 cells during osteogenic differentiation. Relative band integrity was normalized by expression level of β-Actin. The proteins analyzed were BMP-2 (**B**) Osterix (**C**) and Osteocalcin (**D**). Values are expressed as mean ± SEM., ** *p* < 0.01.

To prove that BMP-2 expression could be regulated by PARP activity, protein levels of BMP-2 signaling pathway elements were analyzed during osteogenic differentiation. Following exposure to 1 µM PJ34 during 30 days of osteogenic differentiation, protein levels of BMP-2, Osterix and Osteocalcin were significantly attenuated (Figure 9).

## 3. Discussion

**Figure 10:**
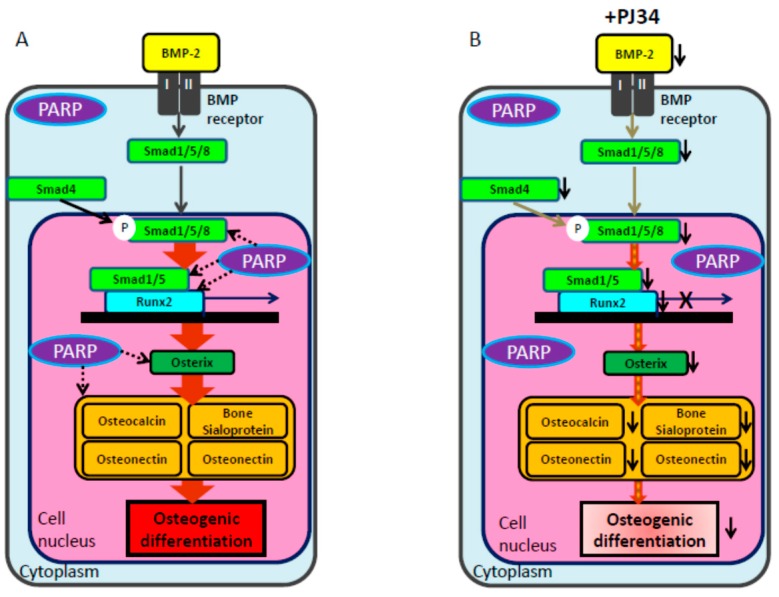
Schema of osteogenic differentiation through the BMP signaling pathway and possible regulation by PARP activity. (**A**) BMP-2 activates Smad1/5/8 (dark gray arrows) and upregulates *Runx2* transcription to promote osteogenic differentiation. Runx2 induces expression of *Osterix* and accelerates transcription of *Osteocalcin*, *Bone Sialoprotein*, *Osteonectin*, and *Osteopontin* (brown thick arrows). PARP activity may also be involved in this pathway (black dotted arrows). Dotted lines are based on the results of this report; (**B**) PJ34 is suggested to suppress BMP-2 and Smad4 signaling (light gray arrows), leading to attenuation of mRNA levels for these factors (diagonal stripe arrows) and subsequent decrease in osteogenic differentiation.

In this study, PARP inhibitor PJ34 delayed and suppressed osteogenic differentiation of BMMSCs and KUSA-A1 cells while chondrogenic and adipogenic differentiation were unaffected, suggesting that PARP activity could be involved in the osteogenic differentiation process especially after commitment into osteoblasts. MSCs have potential to be differentiated into several cell types, including osteoblasts, chondrocytes, adipocytes and so on [22,23,24]. However, regulation of the transcription factors during differentiation is not fully understood. We found that the mRNA expression levels (Figure 7 and Figure 8) and protein expression levels (Figure 9) of the factors involved in BMP-2 signaling pathway in osteogenic differentiation were decreased following exposure to PJ34 suggesting that BMP-2 expression could be regulated by PARP activity (Figure 10).

The role of poly(ADP-ribosyl)ation in the osteogenic differentiation process has not been fully elucidated and only two studies have reported involvement of PARP function in osteogenic differentiation. In the first, 3-Aminobenzamide (3-AB)—one of the first-generation PARP inhibitors that is not used for therapeutic purpose because of its low specificity [25]—was found to cause apoptotic cell death and osteogenic differentiation attenuation in SAOS-2 cells [26]. The second study reported that PAR signaling induced by hydrogen peroxide regulated cell death and osteogenic differentiation in SAOS-2 cells, a feature significantly enhanced by high doses of PJ34, PARP-1-silencing vector or PARG-silencing vector [27]. In general, PJ34 is applied at a dose of 10 µM (or 50 µM) for *in vitro* analysis of both cellular differentiation and cytotoxicity [28,29]. Different from the above studies, the dose of PJ34 used in this study was determined not to induce cytotoxicity based on the results of MTT assay and survival assay.

To confirm inhibition of PARP activity after PJ34 treatment, PAR synthesis was analyzed after hydrogen peroxide stimulation (Figure 3), because we could not detect the synthesis of PAR without any stimulation. Theoretically, the mechanism of PJ34 is competitive blockade of NAD^+^ binding to PARP-1 (to synthesize PAR) [15] and *PARP-1* mRNA levels are not considered to be affected. However, PARP-1 activity was reportedly reduced by 10 µM PJ34 in human colon and liver cancer cells [29]. Unexpectedly, we observed *PARP-1* mRNA levels were significantly reduced by 1 µM PJ34 in KUSA-A1 cells but not in BMMSCs 20 and 30 days after PJ34 treatment (Figure 8E). Therefore, it is suggested that PJ34 could reduce *PARP-1* mRNA expression levels in a dose- and time-dependent manner in KUSA-A1 cells. The effect of PJ34 on PARP-1 has not been fully examined yet during this long duration, however, it is at least indicated that even 1 µM PJ34 could reduce synthesis of PAR and suppress osteogenic differentiation without showing particular cytotoxicity.

Previous reports of PARP family member involvement in MSC differentiation [17,18,19,20], and our current study, suggest that the activity of specific PARP family member(s) promotes osteogenic differentiation via BMP-2 signaling. To further understand how PARP activity is involved in regulation of BMP-2 signaling during osteogenic differentiation, various interacting factors must be investigated—Wnt, hedgehog, Notch and TGF-β signaling—as relationships to poly(ADP-ribosyl)ation or cleavage of PARP have previously been reported in other cell lines [30,31,32,33].

PARP inhibition is currently considered as a novel molecular target drug for cancer therapy [11,14]; however, side effects of available PARP inhibitors have not been fully examined in clinical trials, *in vivo* or *in vitro* studies [16]. The delay and suppression of MSC osteogenic differentiation induced by PJ34 treatment in this study indicate that PARP inhibitors have the potential to impede bone metabolism. Therefore, we suggest that clinical use of PARP inhibitors should be carefully considered, especially for cancer patients with bone metastasis, elderly patients with a high risk of fractures, and pediatric patients whose level of bone metabolism is being monitored. For future understanding for clinical application, it will therefore be necessary to investigate the effects of PARP inhibitor on differentiation and maturation of both osteoblast and osteoclast using *in vivo* models.

## 4. Experimental Section

All animal experimental procedures were approved by the Ethical Committee for Guidelines on Animal Experiments of Tsurumi University School of Dental Medicine on 3 July 2012 (No. 12040).

### 4.1. Culture of Mouse Bone Marrow-Derived Mesenchymal Stem Cells

Two types of cells, mouse bone marrow mesenchymal stem cells (BMMSCs) and mesenchymal progenitor cells (KUSA-A1 cells, RIKEN, Tsukuba, Japan, obtained 30 January 2013), were used in this study. BMMSCs were obtained from 6-week-old male C57BL/6 mice (CLEA, Tokyo, Japan) as previously described [34]. Briefly, BMMSCs were isolated from femurs of 6-week-old male C57BL/6 mice and seeded into culture dishes. After being incubated for 4 h at 37 °C in 5% CO_2_, cells were washed twice with α-Minimum Essential Medium (α-MEM, Wako-Junyaku, Osaka, Japan). Growth medium consisted of α-MEM with 2 mM l-glutamax (Thermo Fisher, Waltham, MA, USA), 20% fetal bovine serum (FBS; Biowest, Nuaillé, France), 100 U/mL penicillin, 100 µg/mL streptomycin and 55 µM 2-mercaptoethanol (Sigma-Aldrich, St. Louis, MO, USA). Primary cultures (passage 0 (P0)) were passaged to disperse colony-forming cells before seeding onto fresh culture dishes (P1). Growth medium was changed every three days and BMMSCs were passed 1:5 upon reaching confluence.

### 4.2. MTT (Microculture Tetrazolium) Assay

BMMSCs and KUSA-A1 cells were plated into 24-well plates at a density of 2.0 × 10^4^ cells/well. After overnight incubation, culture medium was replaced with fresh medium containing various concentrations of either PARP inhibitor PJ34 (Sigma-Aldrich) or AZD2281 (ChemScene, Monmouth Junction, NJ, USA). After being treated for 24 h, the number of viable cells was assessed using a 3-(4-,5-dimethylthiazol-2-yl)-2,5-dyphenyl tetrazolium bromide (MTT; Sigma-Aldrich) assay as previously reported [35]. Briefly, 500 µg of MTT in 100 µL RPMI-1640 Medium (Sigma-Aldrich) was added to each well and incubated for 4 h at 37 °C. After incubation, medium was carefully removed and 200 µL of 0.1 N HCl in isopropanol was added to each well to dissolve resultant formazan crystals. Absorbance was recorded at 570 nm using Microplate Reader Model 680 (Bio-Rad, Hercules, CA, USA) with a 96-well assay plate (Sumilon, Sumitomo Bakelite, Tokyo, Japan). All experiments were performed in triplicate.

IC_50_ was calculated by Excel software (Microsoft, Redmond, Wachington, DC, USA), using the logarithm function. First, the concentration was plotted on the *x*-axis, and cell viability was plotted on the *y*-axis. Then, using the value of higher and lower sides of 50% of concentration and cell viability, a linear equation was created as follows:

IC_50_ = 10^(log(A/B) × (50 − C)/(D − C) + log(B)) (1)

A: the concentration of higher side of 50% of cell viability, B: the concentration of lower side of 50% of cell viability, C: cell viability at the concentration of B, D: cell viability at the concentration of A, ^: symbol of power in Excel software.

### 4.3. Survival Assay

BMMSCs and KUSA-A1 cells were seeded into 12-well plates at a density of 2.0 × 10^3^ cells/well and cultured in growth medium with various concentrations of either PARP inhibitors PJ34 or AZD2281 for 18 h, rinsed two times in phosphate buffered saline (PBS) and allowed to grow. Cells were analyzed when cells cultured without PARP inhibitors reached confluence. Cells were fixed with 4% paraformaldehyde (PFA, Wako-Junyaku), stained with 0.02% crystal violet (Sigma-Aldrich) and the absorbance level was determined at 570 nm with Microplate Reader Model 680.

Suppression of cell survival by 50% was also calculated using the same formula described above.

IC_50_ = 10^(log(A/B) × (50 − C)/(D − C) + log(B)) (2)

A: the concentration of higher side of 50% of absorbance, B: the concentration of lower side of 50% of absorbance, C: reduction rate of absorbance at the concentration of B, D: reduction rate of absorbance at the concentration of A, ^: symbol of power in Excel software.

### 4.4. Proliferation Assay

BMMSCs and KUSA-A1 cells were seeded in triplicate into 6-well plates at a density of 4 × 10^3^ cells/well in triplicate with growth medium containing 0, 1 or 5 µM PJ34. Culture medium was changed every 3 days. Cell numbers were counted by hemocytometer.

### 4.5. Immunocytochemical Detection of Poly(ADP-ribose)

Poly(ADP-ribose) (PAR) synthesis was detected by immunocytochemical analysis as previously described [36] with a slight modification, as follows. BMMSCs and KUSA-A1 cells were seeded onto coverslips (Nunc, Rochester, NY, USA) and incubated with 0, 1 or 5 µM PJ34. Cells were treated with 500 µM hydrogen peroxide for 0, 10, 30 or 60 min, and fixed in ice-cold methanol for 10 min. After washing twice with PBS, cells were blocked in 0.1% bovine serum albumin (BSA; Sigma-Aldrich) diluted in PBS with 0.5% triton X-100 for 15 min. Subsequently, cells were incubated with anti-PAR antibody (1:200 dilution, Abcam, Cambridge, UK) at 4 °C overnight. After several PBS washes, coverslips were incubated with goat anti-chicken IgG (1:300 dilution, Vector Laboratories, Burlingame, CA, USA) at room temperature for 30 min. Bound antibody was visualized using an ABC Elite Detection Kit (Vector Laboratories) and 3,3’-diaminobenzidine substrate according to the manufacturer’s protocol. Coverslips were viewed with BX51 System Microscope (Olympus, Tokyo, Japan) and images were taken using a digital microscope camera (Olympus DP70).

### 4.6. Osteogenic Cell Differentiation and Analysis

Initially, BMMSCs and KUSA-A1 cells were seeded into 12-well plates at a density of 6 × 10^3^ cells/well in growth medium and were cultured to confluence. Medium was then switched to osteogenic differentiation medium comprised of 5 µM ascorbic acid, 1 µM dexamethasone, 1 mM β-glycerophosphoric acid, 100 U/mL penicillin, 100 µg/mL streptomycin, 55 µM 2-mercaptoethanol (all from Sigma-Aldrich) and 2 mM l-glutamax (Thermo Fisher) in α-MEM [37] with or without PARP inhibitor PJ34 at 1 µM (day 0). Medium was freshly changed every 3 days. For analysis of osteogenic differentiation, von Kossa staining and Alizarin Red S staining were performed. Initially, culture medium was removed and cells were washed twice with PBS before fixation in 4% PFA for 25 min. After PFA was removed, cells were washed twice with distilled water and allowed to air dry. For detection of calcium, cells were stained with 1% Alizarin Red S (Sigma-Aldrich) adjusted to pH6.3 by ammonium hydroxide (Wako-Junyaku) for 15 min at 37 °C. Cells were washed twice with distilled water and incubated in 1 mL of 1% HCl in 70% ethanol for 1 h at 4 °C, as previously reported [38]. Solutions (10 µL) were then applied to wells of a 96-well assay plate (Sumilon), and absorbance was determined at 450 nm by Microplate Reader Model 680 (Bio-Rad). For calcium phosphate and calcium carbonate detection, fixed cells were stained with 5% silver nitrate solution (Sigma-Aldrich) dissolved in distilled water for 1 h under UV light, before washing twice with distilled water. To analyze von Kossa staining in a time-course, photos were taken at each time point and digital images were analyzed by ImageJ software (NCI, Bethesda, MD, USA). All images were converted from color to gray scale in order to measure relative density and brightness. Density of crystal violet staining was also measured to confirm assessment of equal cellular densities between samples with or without PJ34 treatment.

### 4.7. Chondrogenic Cell Differentiation and Analysis

In this examination, BMMSCs were solely used because KUSA-A1 cells are mesenchymal progenitor cells, which are unable to differentiate into adipocytes or chondrocytes [21]. BMMSCs were seeded into 12-well plates at a density of 6 × 10^3^ cells/well in growth medium. After cell cultures reached confluence, growth medium was switched to chondrogenic differentiation medium containing 1 µM dexamethasone, 0.17 mM ascorbic acid, 1 ng/mL transforming growth factor-β3 (TGF-β3), 1% insulin-transferrin-selenic acid (ITS) supplement, 100 U/mL penicillin and 100 µg/mL streptomycin in Dulbecco’s modified Eagle’s medium (DMEM; all from Sigma-Aldrich) [39], with or without 1 µM PJ34. For chondrogenic differentiation analysis, cultured cells were fixed and stained with 0.05% Alcian Blue solution (pH 2.5, Wako-Junyaku) to detect glycosaminoglycans. To analyze chondrogenic differentiation levels in a time-course, photos of Alcian Blue staining were taken at each time point and digital images were analyzed by ImageJ software, as previously described.

### 4.8. Adipogenic Cell Differentiation and Analysis

Upon reaching confluence, growth medium was switched to adipogenic differentiation medium comprised of 1 µM dexamethasone, 1 µM insulin, 200 µM indomethacin, 0.5 µM 3-isobutyl-1-methylxanthine, 100 U/mL penicillin, 100 µg/mL streptomycin (all from Sigma-Aldrich) and 10% FBS (Biowest) in DMEM, with or without 1 µM PJ34. After cell fixation in 4% PFA for 25 min, cells were washed twice with 60% isopropanol. For lipid detection, cells were stained with Oil Red O solution (Wako-Junyaku) dissolved in 60% isopropanol for 20 min [40]. To analyze adipogenic differentiation levels over a time-course, photos of Oil Red O staining were taken at each time point and digital images were analyzed by ImageJ software, as previously described.

### 4.9. Total RNA Extraction and Real-Time PCR

Total RNA was isolated from cells by using TRIzol reagent (Invitrogen, Waltham, MA, USA) according to the manufacturer’s protocols. Isolated RNA (5 µg) was reverse transcribed to cDNA by using PrimeScript 1st Strand cDNA Synthesis Kit (Takara, Ohtsu, Shiga, Japan) according to the manufacturer’s protocols. Real-time PCR analyses were performed by using SYBR Premix Ex Taq II (Takara) and the StepOnePlus Real-Time PCR System (Applied Biosystems, Waltham, MA, USA) at recommended thermal cycling settings: one initial cycle at 95 °C for 30 s, followed by 40 cycles at 95 °C for 3 s and 60 °C for 30 s. Primer details are summarized in Table 1. *GAPDH* was used as an internal control and quantitative gene expression was normalized to *GAPDH* expression level.

**Table 1:** Sequence of primers used in real-time RT-PCR.

Gene	Length	Forward Sequence (5′>3′)	Reverse Sequence (5′>3′)
*Runx2*	250 bp	CCACCACTCACTACCACACG	TCAGCGTCAACACCATCATT
*Osterix*	373 bp	ACCAGGTCCAGGCAACACACCTAC	GCAGTCGCAGGTAGAACGCCCTGC
*BMP2*	154 bp	GGGACCCGCTGTCTTCTAGT	TCAACTCAAATTCGCTGAGGAC
*Osteocalcin*	302 bp	AGACAAGTCCCACACAGCAG	GGCGGTCTTCAAGCCATACT
*Bone sialoproiten*	312 bp	CTGAAGCAGGTGCAGAAGGA	TCTGACCCTCGTAGCCTTCA
*Osteopontin*	354 bp	CTGGCAGCTCAGAGGAGAAG	GGACATCGACTGTAGGGACG
*ALP*	211 bp	TAACACCAACGCTCAGGTCC	GTGGTTCACCCGAGTGGTAG
*Smad1*	97 bp	ACCTGTGGCTTCCGTCTC	ATCGTGGCTCCTTCGTC
*Smad4*	145 bp	CAGCACTACCACCTGGACTGGA	CTGGAATGCAAGCTCATTGTGAA
*Smad5*	197 bp	AAGTAGATTCTGCCTGGGATT	AGACGGTGGTGGGATGG
*Smad8*	169 bp	ATCCCTGGCAATCTGTA	CCCTGGCTGTCCTGTAA
*PARP-1*	72 bp	GGAAAGGGATCTACTTTGCCG	TCGGGTCTCCCTGAGATGTG
*GAPDH*	240 bp	TGATGACATCAAGAAGGTGGTGAAG	TCCTTGGAGGCCATGTAGGCCAT

### 4.10. Western Blot Analysis

BMMSCs and KUSA-A1 cells were lysed with RIPA buffer (10 mM Tris–HCl, 1% NP-40, 0.1% SDS, 150 mM NaCl and 1 mM EDTA) containing protease inhibitor cocktail (Thermo Fisher), and each sample was centrifuged at 10,000× *g*, 4 °C for 20 min. NuPAGE LDS sample buffer (Invitrogen) was added to sample supernatant, subsequently samples were heated at 98 °C for 5 min. Samples (80 µg protein) were separated electrophoretically by the NuPAGE System with 12% Bis-Tris gel and electroblotted onto a polyvinylidene difluoride membrane by using an iBlot Dry Blotting System (all from Invitrogen). The membrane was blocked with 5% skim milk (Wako-Junyaku) at room temperature for 30 min, before primary antibody incubations were performed in 2.5% skim milk at 4 °C overnight. Antibodies used in this study were anti-BMP-2 (1:500 dilution, Bioss Antibodies, Woburn, MA, USA), anti-Osterix (1:1000 dilution, Bioss Antibodies), anti-Osteocalcin (1:500 dilution, Abcam) and anti-β-Actin (1:1000 dilution, Santa Cruz Biotechnology, Santa Cruz, CA, USA). Membranes were subsequently incubated with peroxidase-conjugated secondary antibody at room temperature for 30 min. Specific bands were detected with a chemiluminescence assay (Amersham ECL Prime Western Blotting Detection Reagent, GE Healthcare, Buckinghamshire, UK). Then, images were scanned by C-DiGit Scanner and analyzed using Image Studio Lite Software (both from Li-COR Biosciences, Lincoln, NE, USA).

### 4.11. Statistical Analysis

Group comparisons were undertaken using an independent *t*-test. All statistical analyses were performed using SPSS v.20.0 (IBM Corp., Armonk, NY, USA).

## 5. Conclusions

In this study, osteogenic differentiation of BMMSCs and KUSA-A1 cells was suppressed after treatment with 1 µM PARP inhibitor PJ34 without showing cytotoxic effects, and the mRNA and protein expression levels of the factors involved in BMP-2 signaling pathway were suppressed. On the contrary, chondrogenic and adipogenic differentiation of BMMSCs was not significantly affected. Therefore, the current *in vitro* study suggests that poly(ADP-ribosyl)ation could be involved in osteogenic differentiation through the BMP-2 signaling pathway. Moreover, our results also suggest that PJ34 decreases bone metabolism, indicating a heightened need for careful indication of PARP inhibitors for cancer patients whose bone metabolism levels are being monitored.

## Supplementary Materials

Supplementary materials can be found at http://www.mdpi.com/1422-0067/16/10/24820/s1.

## References

[1] Huang W., Yang S., Shao J., Li Y.P. (2007). Signaling and transcriptional regulation in osteoblast commitment and differentiation. Front. Biosci..

[2] Fakhry M., Hamade E., Badran B., Buchet R., Magne D. (2013). Molecular mechanisms of mesenchymal stem cell differentiation towards osteoblasts. World J. Stem Cells.

[3] Chen G., Deng C., Li Y.P. (2012). TGF-β and BMP signaling in osteoblast differentiation and bone formation. Int. J. Biol. Sci..

[4] Phimphilai M., Zhao Z., Boules H., Roca H., Franceschi R.T. (2006). BMP signaling is required for RUNX2-dependent induction of the osteoblast phenotype. J. Bone Miner. Res..

[5] Malanga M., Pleschke J.M., Kleczkowska H.E., Althaus F.R. (1998). Poly(ADP-ribose) binds to specific domains of p53 and alters its DNA binding functions. J. Biol. Chem..

[6] Pleschke J.M., Kleczkowska H.E., Strohm M., Althaus F.R. (2000). Poly(ADP-ribose) binds to specific domains in DNA damage checkpoint proteins. J. Biol. Chem..

[7] Morales J., Li L., Fattah F.J., Dong Y., Bey E.A., Patel M., Gao J., Boothman D.A. (2014). Review of poly(ADP-ribose) polymerase (PARP) mechanisms of action and rationale for targeting in cancer and other diseases. Crit. Rev. Eukaryot. Gene Expr..

[8] Seimiya H. (2006). The telomeric PARP, tankyrases, as targets for cancer therapy. Br. J. Cancer.

[9] Masutani M., Fujimori H. (2013). Poly(ADP-ribosyl)ation in carcinogenesis. Mol. Asp. Med..

[10] Masutani M., Nozaki T., Nishiyama E., Shimokawa T., Tachi Y., Suzuki H., Nakagama H., Wakabayashi K., Sugimura T. (1999). Function of poly(ADP-ribose) polymerase in response to DNA damage: Gene-disruption study in mice. Mol. Cell. Biochem..

[11] Tahara M., Inoue T., Sato F., Miyakura Y., Horie H., Yasuda Y., Fujii H., Kotake K., Sugano K. (2014). The use of olaparib (AZD2281) potentiates SN-38 cytotoxicity in colon cancer cells by indirect inhibition of Rad51-mediated repair of DNA double-strand breaks. Mol. Cancer Ther..

[12] Ratnam K., Low J.A. (2007). Current development of clinical inhibitors of poly(ADP-ribose) polymerase in oncology. Clin. Cancer Res..

[13] Del Conte G., Sessa C., von Moos R., Vigano L., Digena T., Locatelli A., Gallerani E., Fasolo A., Tessari A., Cathomas R. (2014). Phase I study of olaparib in combination with liposomal doxorubicin in patients with advanced solid tumours. Br. J. Cancer.

[14] Liu J.F., Tolaney S.M., Birrer M., Fleming G.F., Buss M.K., Dahlberg S.E., Lee H., Whalen C., Tyburski K., Winer E. (2013). A phase I trial of the poly(ADP-ribose) polymerase inhibitor olaparib (AZD2281) in combination with the anti-angiogenic cediranib (AZD2171) in recurrent epithelial ovarian or triple-negative breast cancer. Eur. J. Cancer.

[15] Steffen J.D., Brody J.R., Armen R.S., Pascal J.M. (2013). Structural implications for selective targeting of PARPS. Front. Oncol..

[16] Fong P.C., Boss D.S., Yap T.A., Tutt A., Wu P., Mergui-Roelvink M., Mortimer P., Swaisland H., Lau A., O’Connor M.J. (2009). Inhibition of poly(ADP-ribose) polymerase in tumors from BRCA mutation carriers. N. Engl. J. Med..

[17] Bai P., Houten S.M., Huber A., Schreiber V., Watanabe M., Kiss B., de Murcia G., Auwerx J., Menissier-de Murcia J. (2007). Poly(ADP-ribose) polymerase-2 controls adipocyte differentiation and adipose tissue function through the regulation of the activity of the retinoid X receptor/peroxisome proliferator-activated receptor-gamma heterodimer. J. Biol. Chem..

[18] Gilbert L., He X., Farmer P., Boden S., Kozlowski M., Rubin J., Nanes M.S. (2000). Inhibition of osteoblast differentiation by tumor necrosis factor-α. Endocrinology.

[19] Majewski P.M., Thurston R.D., Ramalingam R., Kiela P.R., Ghishan F.K. (2010). Cooperative role of NF-κB and poly(ADP-ribose) polymerase 1 (PARP-1) in the TNF-induced inhibition of PHEX expression in osteoblasts. J. Biol. Chem..

[20] Wang C.Y., Chen L.L., Kuo P.Y., Chang J.L., Wang Y.J., Hung S.C. (2010). Apoptosis in chondrogenesis of human mesenchymal stem cells: Effect of serum and medium supplements. Apoptosis.

[21] Higuchi A., Shindo Y., Gomei Y., Mori T., Uyama T., Umezawa A. (2005). Cell separation between mesenchymal progenitor cells through porous polymeric membranes. J. Biomed. Mater. Res. B Appl. Biomater..

[22] Dominici M., Le Blanc K., Mueller I., Slaper-Cortenbach I., Marini F., Krause D., Deans R., Keating A., Prockop D., Horwitz E. (2006). Minimal criteria for defining multipotent mesenchymal stromal cells. The international society for cellular therapy position statement. Cytotherapy.

[23] Mohammadian M., Shamsasenjan K., Lotfi Nezhad P., Talebi M., Jahedi M., Nickkhah H., Minayi N., Movassagh Pour A. (2013). Mesenchymal stem cells: New aspect in cell-based regenerative therapy. Adv. Pharm. Bull..

[24] Zhang Y., Khan D., Delling J., Tobiasch E. (2012). Mechanisms underlying the osteo- and adipo-differentiation of human mesenchymal stem cells. Sci. World J..

[25] Peralta-Leal A., Rodriguez-Vargas J.M., Aguilar-Quesada R., Rodriguez M.I., Linares J.L., de Almodovar M.R., Oliver F.J. (2009). Parp inhibitors: New partners in the therapy of cancer and inflammatory diseases. Free Radic. Biol. Med..

[26] De Blasio A., Musmeci M.T., Giuliano M., Lauricella M., Emanuele S., D’Anneo A., Vassallo B., Tesoriere G., Vento R. (2003). The effect of 3-aminobenzamide, inhibitor of poly(ADP-ribose) polymerase, on human osteosarcoma cells. Int. J. Oncol..

[27] Robaszkiewicz A., Erdelyi K., Kovacs K., Kovacs I., Bai P., Rajnavolgyi E., Virag L. (2012). Hydrogen peroxide-induced poly(ADP-ribosyl)ation regulates osteogenic differentiation-associated cell death. Free Radic. Biol. Med..

[28] Madison D.L., Stauffer D., Lundblad J.R. (2011). The PARP inhibitor PJ34 causes a PARP1-independent, p21 dependent mitotic arrest. DNA Repair.

[29] Jouan-Lanhouet S., Arshad M.I., Piquet-Pellorce C., Martin-Chouly C., Le Moigne-Muller G., Van Herreweghe F., Takahashi N., Sergent O., Lagadic-Gossmann D., Vandenabeele P. (2012). Trail induces necroptosis involving RIPK1/RIPK3-dependent PARP-1 activation. Cell Death Differ..

[30] Fearon E.R. (2009). Parsing the phrase “all in for Axin”—Wnt pathway targets in cancer. Cancer Cell..

[31] Nanta R., Kumar D., Meeker D., Rodova M., Van Veldhuizen P.J., Shankar S., Srivastava R.K. (2013). NVP-LDE-225 (Erismodegib) inhibits epithelial-mesenchymal transition and human prostate cancer stem cell growth in NOD/SCID IL2Rγ null mice by regulating Bmi-1 and microRNA-128. Oncogenesis.

[32] Yang J., Zhang W. (2013). New molecular insights into osteosarcoma targeted therapy. Curr. Opin. Oncol..

[33] Lonn P., van der Heide L.P., Dahl M., Hellman U., Heldin C.H., Moustakas A. (2010). PARP-1 attenuates Smad-mediated transcription. Mol. Cell.

[34] Miura M., Miura Y., Padilla-Nash H.M., Molinolo A.A., Fu B., Patel V., Seo B.M., Sonoyama W., Zheng J.J., Baker C.C. (2006). Accumulated chromosomal instability in murine bone marrow mesenchymal stem cells leads to malignant transformation. Stem Cells.

[35] Li M., Zhao L., Liu J., Liu A.L., Zeng W.S., Luo S.Q., Bai X.C. (2009). Hydrogen peroxide induces G2 cell cycle arrest and inhibits cell proliferation in osteoblasts. Anat. Rec..

[36] Burkle A., Chen G., Kupper J.H., Grube K., Zeller W.J. (1993). Increased poly(ADP-ribosyl)ation in intact cells by cisplatin treatment. Carcinogenesis.

[37] Sila-Asna M., Bunyaratvej A., Maeda S., Kitaguchi H., Bunyaratavej N. (2007). Osteoblast differentiation and bone formation gene expression in strontium-inducing bone marrow mesenchymal stem cell. Kobe J. Med. Sci..

[38] Satomura K., Tobiume S., Tokuyama R., Yamasaki Y., Kudoh K., Maeda E., Nagayama M. (2007). Melatonin at pharmacological doses enhances human osteoblastic differentiation *in vitro* and promotes mouse cortical bone formation *in vivo*. J. Pineal Res..

[39] Bouffi C., Thomas O., Bony C., Giteau A., Venier-Julienne M.C., Jorgensen C., Montero-Menei C., Noel D. (2010). The role of pharmacologically active microcarriers releasing TGF-β3 in cartilage formation *in vivo* by mesenchymal stem cells. Biomaterials.

[40] Cunha M.C., Lima Fda S., Vinolo M.A., Hastreiter A., Curi R., Borelli P., Fock R.A. (2013). Protein malnutrition induces bone marrow mesenchymal stem cells commitment to adipogenic differentiation leading to hematopoietic failure. PLoS ONE.

