# Supplementary material for: PARP Inhibitor PJ34 Suppresses Osteogenic Differentiation in Mouse Mesenchymal Stem Cells by Modulating BMP-2 Signaling Pathway
Source: Int J Mol Sci. 2015 Oct 19;16(10):24820–38. doi: 10.3390/ijms161024820 (PMC4632778; doi:10.3390/ijms161024820)
Supplement: Supplementary file 1 [file ijms-16-24820-s001.pdf]

Supplementary Information

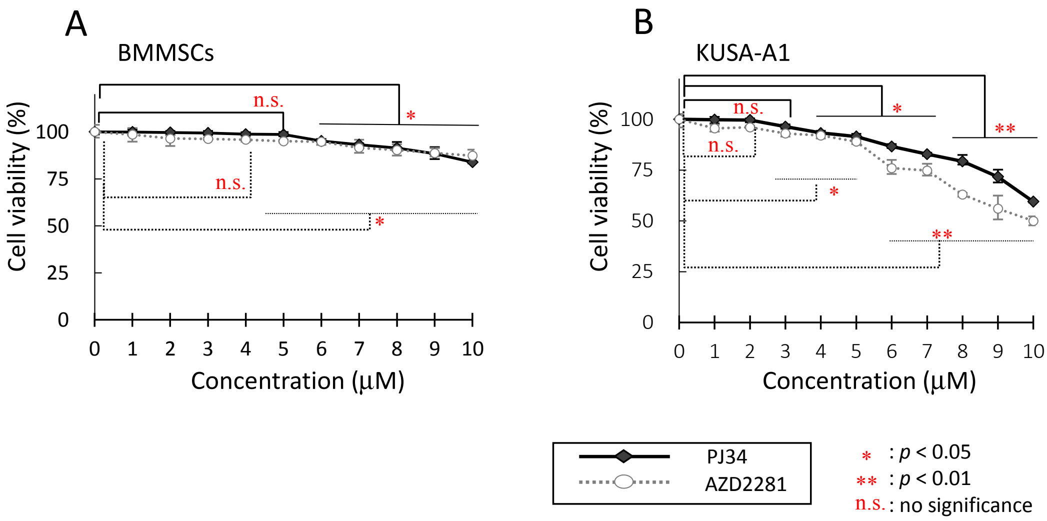

Figure S1. Microculture Tetrazolium Assay (MTT) assay was performed to analyze cytotoxicity of PJ34 and AZD2281 on BMMSCs (**A**) and KUSA-A1 cells (**B**). Values are expressed as the mean ± SEM. * $p < 0.05$, ** $p < 0.01$. n.s. = no significance
